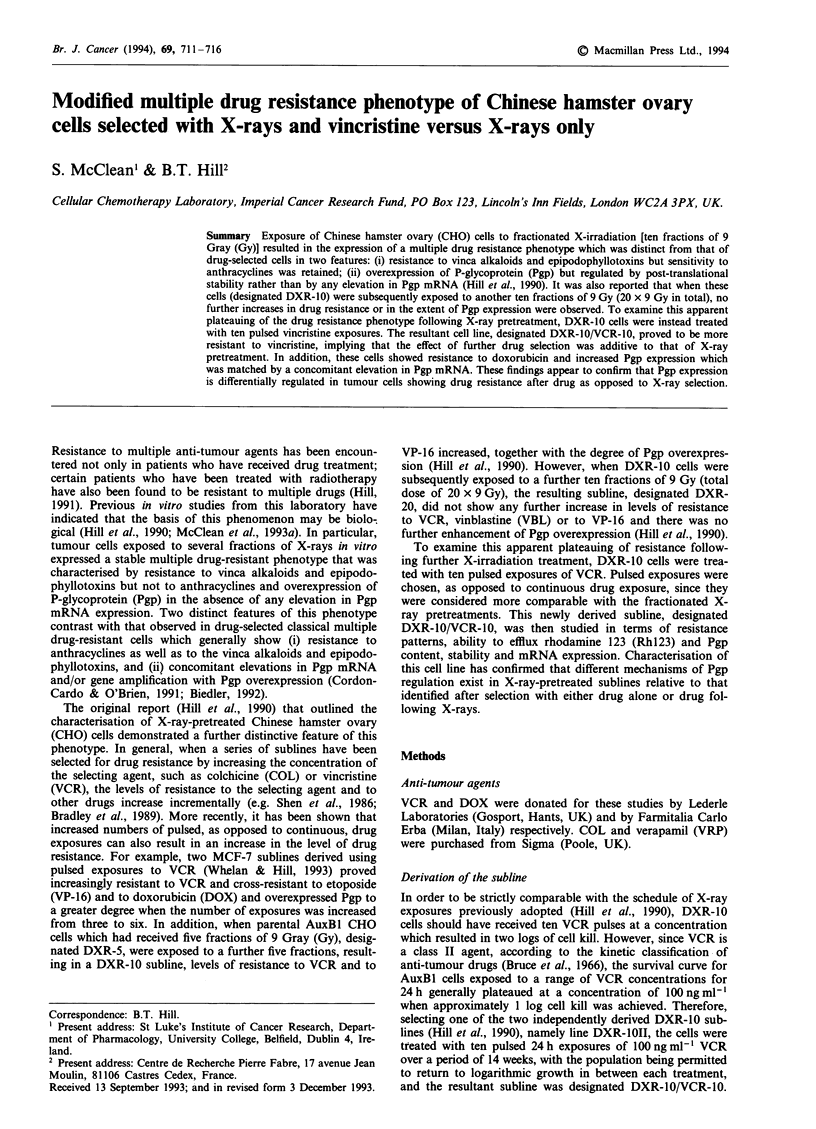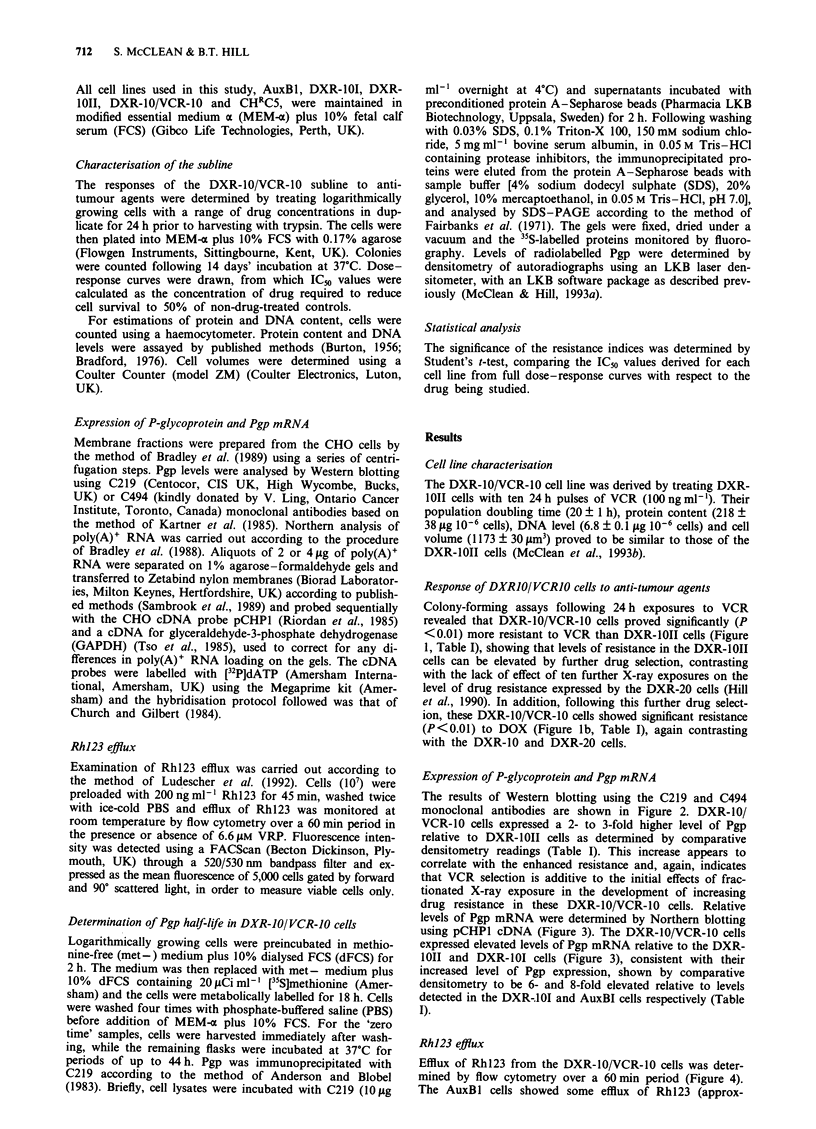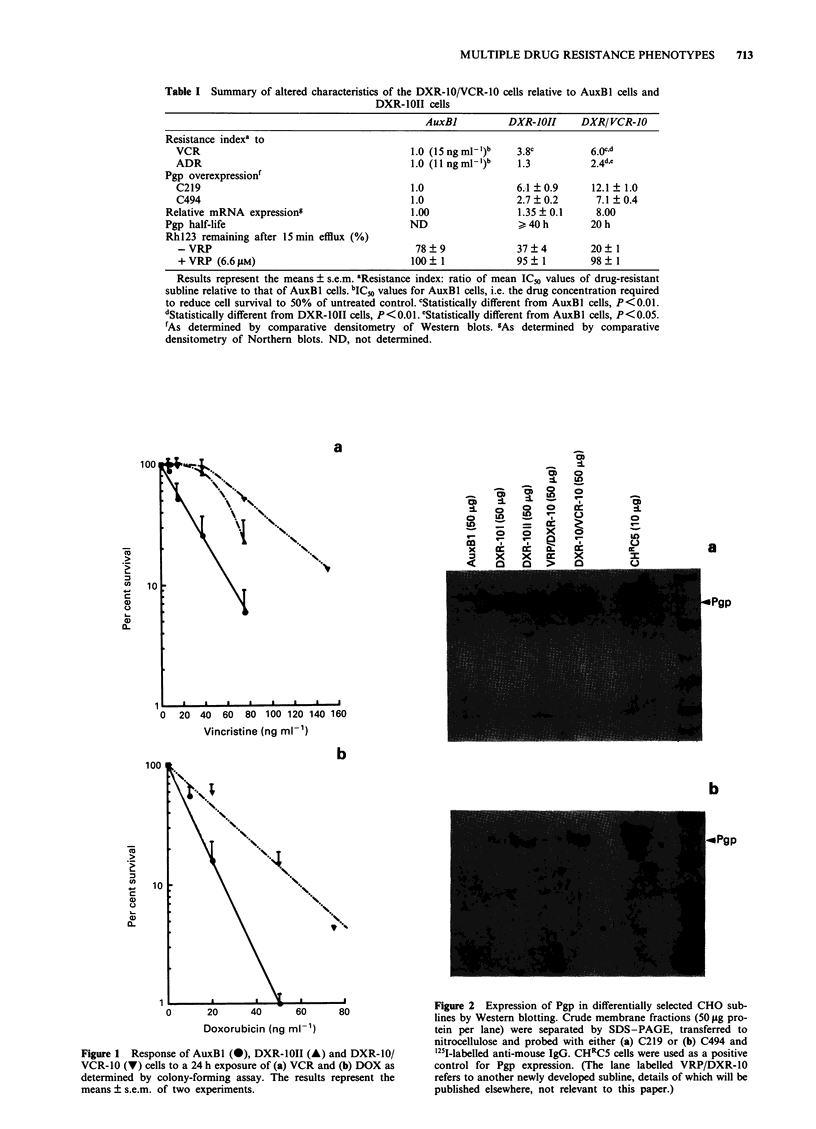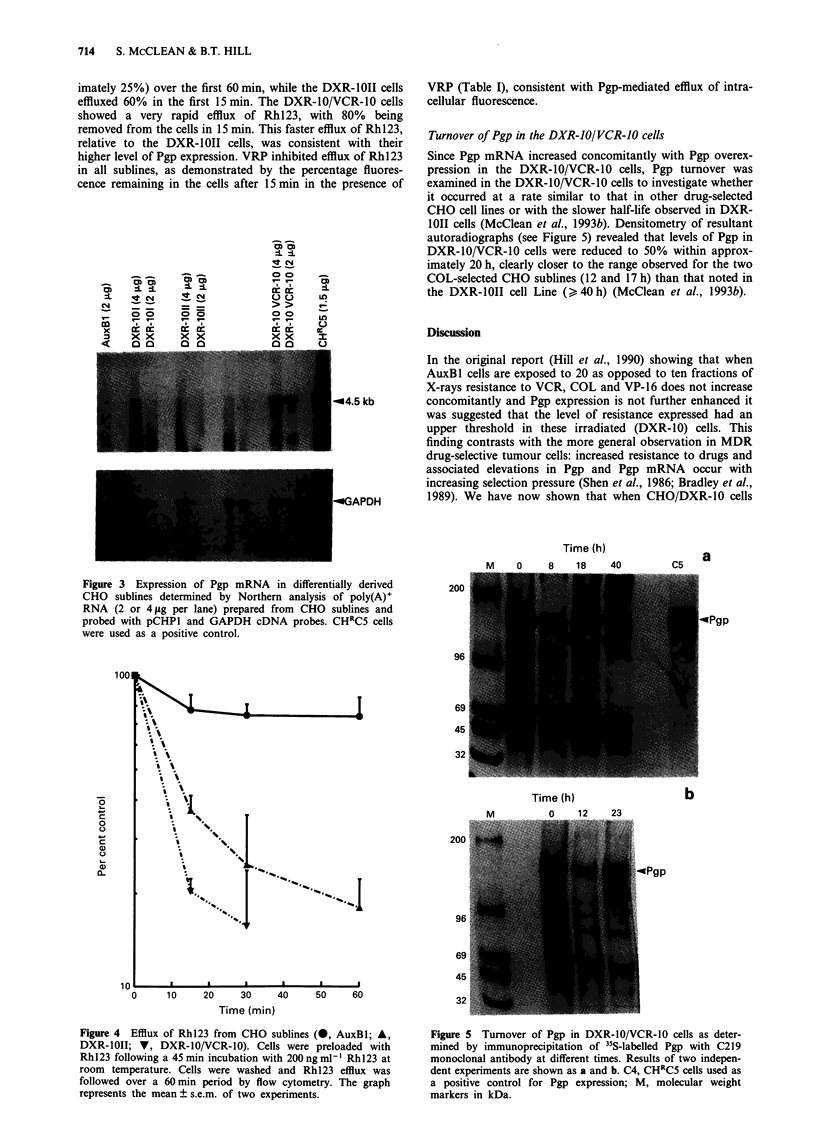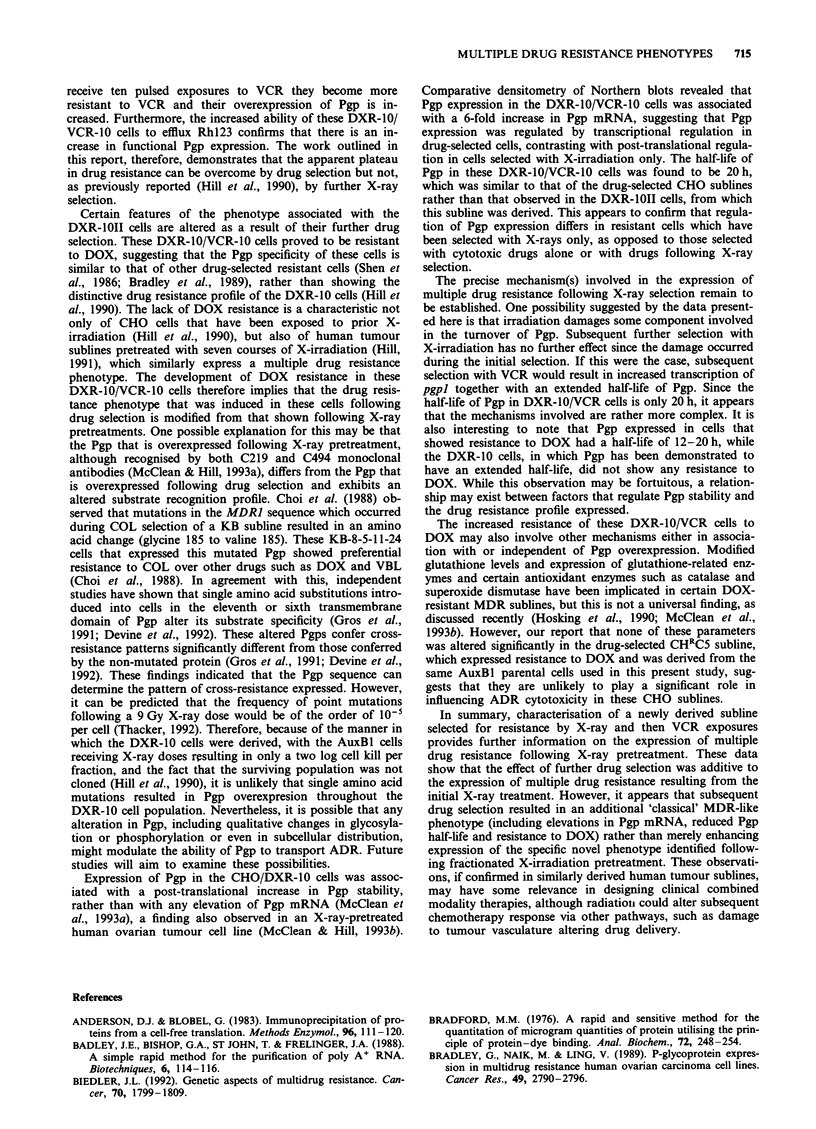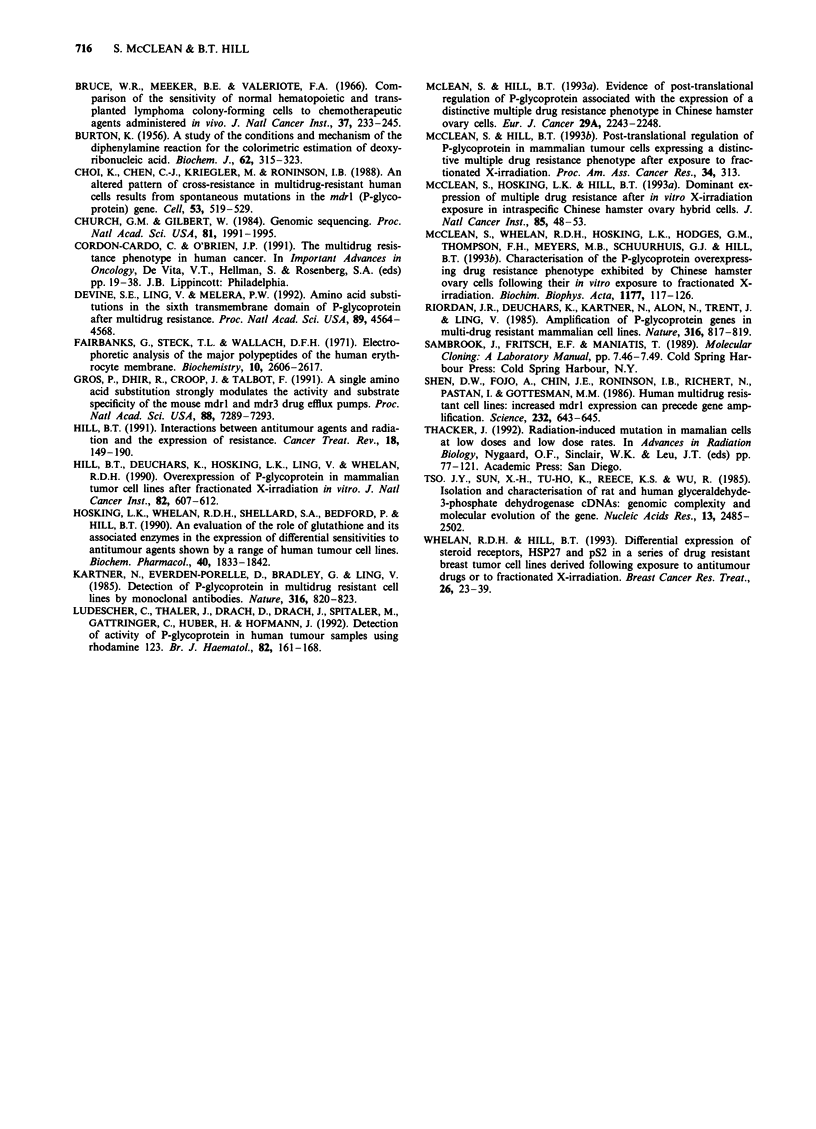# Modified multiple drug resistance phenotype of Chinese hamster ovary cells selected with X-rays and vincristine versus X-rays only.

**DOI:** 10.1038/bjc.1994.134

**Published:** 1994-04

**Authors:** S. McClean, B. T. Hill

**Affiliations:** Cellular Chemotherapy Laboratory, Imperial Cancer Research Fund, London, UK.

## Abstract

**Images:**


Br. J. Cancer (1994), 69, 711-716 © Macmillan Press Ltd., 1994

### Modified multiple drug resistance phenotype of Chinese hamster ovary cells selected with X-rays and vincristine versus X-rays only

S. McClean[1] & B.T. Hill[2]

*Cellular Chemotherapy Laboratory, Imperial Cancer Research Fund, PO Box 123, Lincoln's Inn Fields, London WC2A 3PX, UK.*

**Summary** Exposure of Chinese hamster ovary (CHO) cells to fractionated X-irradiation [ten fractions of 9 Gray (Gy)] resulted in the expression of a multiple drug resistance phenotype which was distinct from that of drug-selected cells in two features: (i) resistance to vinca alkaloids and epipodophyllotoxins but sensitivity to anthracyclines was retained; (ii) overexpression of P-glycoprotein (Pgp) but regulated by post-translational stability rather than by any elevation in Pgp mRNA (Hill *et al.*, 1990). It was also reported that when these cells (designated DXR-10) were subsequently exposed to another ten fractions of 9 Gy (20 × 9 Gy in total), no further increases in drug resistance or in the extent of Pgp expression were observed. To examine this apparent plateauing of the drug resistance phenotype following X-ray pretreatment, DXR-10 cells were instead treated with ten pulsed vincristine exposures. The resultant cell line, designated DXR-10/VCR-10, proved to be more resistant to vincristine, implying that the effect of further drug selection was additive to that of X-ray pretreatment. In addition, these cells showed resistance to doxorubicin and increased Pgp expression which was matched by a concomitant elevation in Pgp mRNA. These findings appear to confirm that Pgp expression is differentially regulated in tumour cells showing drug resistance after drug as opposed to X-ray selection.

Resistance to multiple anti-tumour agents has been encountered not only in patients who have received drug treatment; certain patients who have been treated with radiotherapy have also been found to be resistant to multiple drugs (Hill, 1991). Previous *in vitro* studies from this laboratory have indicated that the basis of this phenomenon may be biological (Hill *et al.*, 1990; McClean *et al.*, 1993*a*). In particular, tumour cells exposed to several fractions of X-rays *in vitro* expressed a stable multiple drug-resistant phenotype that was characterised by resistance to vinca alkaloids and epipodophyllotoxins but not to anthracyclines and overexpression of P-glycoprotein (Pgp) in the absence of any elevation in Pgp mRNA expression. Two distinct features of this phenotype contrast with that observed in drug-selected classical multiple drug-resistant cells which generally show (i) resistance to anthracyclines as well as to the vinca alkaloids and epipodophyllotoxins, and (ii) concomitant elevations in Pgp mRNA and/or gene amplification with Pgp overexpression (Cordon-Cardo & O'Brien, 1991; Biedler, 1992).

The original report (Hill *et al.*, 1990) that outlined the characterisation of X-ray-pretreated Chinese hamster ovary (CHO) cells demonstrated a further distinctive feature of this phenotype. In general, when a series of sublines have been selected for drug resistance by increasing the concentration of the selecting agent, such as colchicine (COL) or vincristine (VCR), the levels of resistance to the selecting agent and to other drugs increase incrementally (e.g. Shen *et al.*, 1986; Bradley *et al.*, 1989). More recently, it has been shown that increased numbers of pulsed, as opposed to continuous, drug exposures can also result in an increase in the level of drug resistance. For example, two MCF-7 sublines derived using pulsed exposures to VCR (Whelan & Hill, 1993) proved increasingly resistant to VCR and cross-resistant to etoposide (VP-16) and to doxorubicin (DOX) and overexpressed Pgp to a greater degree when the number of exposures was increased from three to six. In addition, when parental AuxB1 CHO cells which had received five fractions of 9 Gray (Gy), designated DXR-5, were exposed to a further five fractions, resulting in a DXR-10 subline, levels of resistance to VCR and to VP-16 increased, together with the degree of Pgp overexpression (Hill *et al.*, 1990). However, when DXR-10 cells were subsequently exposed to a further ten fractions of 9 Gy (total dose of 20 × 9 Gy), the resulting subline, designated DXR-20, did not show any further increase in levels of resistance to VCR, vinblastine (VBL) or to VP-16 and there was no further enhancement of Pgp overexpression (Hill *et al.*, 1990).

To examine this apparent plateauing of resistance following further X-irradiation treatment, DXR-10 cells were treated with ten pulsed exposures of VCR. Pulsed exposures were chosen, as opposed to continuous drug exposure, since they were considered more comparable with the fractionated X-ray pretreatments. This newly derived subline, designated DXR-10/VCR-10, was then studied in terms of resistance patterns, ability to efflux rhodamine 123 (Rh123) and Pgp content, stability and mRNA expression. Characterisation of this cell line has confirmed that different mechanisms of Pgp regulation exist in X-ray-pretreated sublines relative to that identified after selection with either drug alone or drug following X-rays.

### Methods

### *Anti-tumour agents*

VCR and DOX were donated for these studies by Lederle Laboratories (Gosport, Hants, UK) and by Farmitalia Carlo Erba (Milan, Italy) respectively. COL and verapamil (VRP) were purchased from Sigma (Poole, UK).

### *Derivation of the subline*

In order to be strictly comparable with the schedule of X-ray exposures previously adopted (Hill *et al.*, 1990), DXR-10 cells should have received ten VCR pulses at a concentration which resulted in two logs of cell kill. However, since VCR is a class II agent, according to the kinetic classification of anti-tumour drugs (Bruce *et al.*, 1966), the survival curve for AuxB1 cells exposed to a range of VCR concentrations for 24 h generally plateaued at a concentration of 100 ng $ml^{-1}$ when approximately 1 log cell kill was achieved. Therefore, selecting one of the two independently derived DXR-10 sublines (Hill *et al.*, 1990), namely line DXR-10II, the cells were treated with ten pulsed 24 h exposures of 100 ng $ml^{-1}$ VCR over a period of 14 weeks, with the population being permitted to return to logarithmic growth in between each treatment, and the resultant subline was designated DXR-10/VCR-10.

Correspondence: B.T. Hill.
[1] Present address: St Luke's Institute of Cancer Research, Department of Pharmacology, University College, Belfield, Dublin 4, Ireland.
[2] Present address: Centre de Recherche Pierre Fabre, 17 avenue Jean Moulin, 81106 Castres Cedex, France.
Received 13 September 1993; and in revised form 3 December 1993.

All cell lines used in this study, AuxB1, DXR-10I, DXR-10II, DXR-10/VCR-10 and $CH^RC5$, were maintained in modified essential medium α (MEM-α) plus 10% fetal calf serum (FCS) (Gibco Life Technologies, Perth, UK).

*Characterisation of the subline*

The responses of the DXR-10/VCR-10 subline to anti-tumour agents were determined by treating logarithmically growing cells with a range of drug concentrations in duplicate for 24 h prior to harvesting with trypsin. The cells were then plated into MEM-α plus 10% FCS with 0.17% agarose (Flowgen Instruments, Sittingbourne, Kent, UK). Colonies were counted following 14 days' incubation at 37°C. Dose–response curves were drawn, from which $IC_{50}$ values were calculated as the concentration of drug required to reduce cell survival to 50% of non-drug-treated controls.

For estimations of protein and DNA content, cells were counted using a haemocytometer. Protein content and DNA levels were assayed by published methods (Burton, 1956; Bradford, 1976). Cell volumes were determined using a Coulter Counter (model ZM) (Coulter Electronics, Luton, UK).

*Expression of P-glycoprotein and Pgp mRNA*

Membrane fractions were prepared from the CHO cells by the method of Bradley *et al.* (1989) using a series of centrifugation steps. Pgp levels were analysed by Western blotting using C219 (Centocor, CIS UK, High Wycombe, Bucks, UK) or C494 (kindly donated by V. Ling, Ontario Cancer Institute, Toronto, Canada) monoclonal antibodies based on the method of Kartner *et al.* (1985). Northern analysis of $poly(A)^+$ RNA was carried out according to the procedure of Bradley *et al.* (1988). Aliquots of 2 or 4 μg of $poly(A)^+$ RNA were separated on 1% agarose–formaldehyde gels and transferred to Zetabind nylon membranes (Biorad Laboratories, Milton Keynes, Hertfordshire, UK) according to published methods (Sambrook *et al.*, 1989) and probed sequentially with the CHO cDNA probe pCHP1 (Riordan *et al.*, 1985) and a cDNA for glyceraldehyde-3-phosphate dehydrogenase (GAPDH) (Tso *et al.*, 1985), used to correct for any differences in $poly(A)^+$ RNA loading on the gels. The cDNA probes were labelled with $[^{32}P]$dATP (Amersham International, Amersham, UK) using the Megaprime kit (Amersham) and the hybridisation protocol followed was that of Church and Gilbert (1984).

*Rh123 efflux*

Examination of Rh123 efflux was carried out according to the method of Ludescher *et al.* (1992). Cells ($10^7$) were preloaded with 200 ng $ml^{-1}$ Rh123 for 45 min, washed twice with ice-cold PBS and efflux of Rh123 was monitored at room temperature by flow cytometry over a 60 min period in the presence or absence of 6.6 μM VRP. Fluorescence intensity was detected using a FACScan (Becton Dickinson, Plymouth, UK) through a 520/530 nm bandpass filter and expressed as the mean fluorescence of 5,000 cells gated by forward and 90° scattered light, in order to measure viable cells only.

*Determination of Pgp half-life in DXR-10/VCR-10 cells*

Logarithmically growing cells were preincubated in methionine-free (met−) medium plus 10% dialysed FCS (dFCS) for 2 h. The medium was then replaced with met− medium plus 10% dFCS containing 20 μCi $ml^{-1}$ $[^{35}S]$methionine (Amersham) and the cells were metabolically labelled for 18 h. Cells were washed four times with phosphate-buffered saline (PBS) before addition of MEM-α plus 10% FCS. For the 'zero time' samples, cells were harvested immediately after washing, while the remaining flasks were incubated at 37°C for periods of up to 44 h. Pgp was immunoprecipitated with C219 according to the method of Anderson and Blobel (1983). Briefly, cell lysates were incubated with C219 (10 μg $ml^{-1}$ overnight at 4°C) and supernatants incubated with preconditioned protein A–Sepharose beads (Pharmacia LKB Biotechnology, Uppsala, Sweden) for 2 h. Following washing with 0.03% SDS, 0.1% Triton-X 100, 150 mM sodium chloride, 5 mg $ml^{-1}$ bovine serum albumin, in 0.05 M Tris–HCl containing protease inhibitors, the immunoprecipitated proteins were eluted from the protein A–Sepharose beads with sample buffer [4% sodium dodecyl sulphate (SDS), 20% glycerol, 10% mercaptoethanol, in 0.05 M Tris–HCl, pH 7.0], and analysed by SDS–PAGE according to the method of Fairbanks *et al.* (1971). The gels were fixed, dried under a vacuum and the $^{35}S$-labelled proteins monitored by fluorography. Levels of radiolabelled Pgp were determined by densitometry of autoradiographs using an LKB laser densitometer, with an LKB software package as described previously (McClean & Hill, 1993*a*).

*Statistical analysis*

The significance of the resistance indices was determined by Student's *t*-test, comparing the $IC_{50}$ values derived for each cell line from full dose–response curves with respect to the drug being studied.

### Results

*Cell line characterisation*

The DXR-10/VCR-10 cell line was derived by treating DXR-10II cells with ten 24 h pulses of VCR (100 ng $ml^{-1}$). Their population doubling time (20 ± 1 h), protein content (218 ± 38 μg $10^{-6}$ cells), DNA level (6.8 ± 0.1 μg $10^{-6}$ cells) and cell volume (1173 ± 30 $\mu m^3$) proved to be similar to those of the DXR-10II cells (McClean *et al.*, 1993*b*).

*Response of DXR10/VCR10 cells to anti-tumour agents*

Colony-forming assays following 24 h exposures to VCR revealed that DXR-10/VCR-10 cells proved significantly ($P < 0.01$) more resistant to VCR than DXR-10II cells (Figure 1, Table I), showing that levels of resistance in the DXR-10II cells can be elevated by further drug selection, contrasting with the lack of effect of ten further X-ray exposures on the level of drug resistance expressed by the DXR-20 cells (Hill *et al.*, 1990). In addition, following this further drug selection, these DXR-10/VCR-10 cells showed significant resistance ($P < 0.01$) to DOX (Figure 1b, Table I), again contrasting with the DXR-10 and DXR-20 cells.

*Expression of P-glycoprotein and Pgp mRNA*

The results of Western blotting using the C219 and C494 monoclonal antibodies are shown in Figure 2. DXR-10/VCR-10 cells expressed a 2- to 3-fold higher level of Pgp relative to DXR-10II cells as determined by comparative densitometry readings (Table I). This increase appears to correlate with the enhanced resistance and, again, indicates that VCR selection is additive to the initial effects of fractionated X-ray exposure in the development of increasing drug resistance in these DXR-10/VCR-10 cells. Relative levels of Pgp mRNA were determined by Northern blotting using pCHP1 cDNA (Figure 3). The DXR-10/VCR-10 cells expressed elevated levels of Pgp mRNA relative to the DXR-10II and DXR-10I cells (Figure 3), consistent with their increased level of Pgp expression, shown by comparative densitometry to be 6- and 8-fold elevated relative to levels detected in the DXR-10I and AuxBI cells respectively (Table I).

*Rh123 efflux*

Efflux of Rh123 from the DXR-10/VCR-10 cells was determined by flow cytometry over a 60 min period (Figure 4). The AuxB1 cells showed some efflux of Rh123 (approx-

**Table I** Summary of altered characteristics of the DXR-10/VCR-10 cells relative to AuxB1 cells and DXR-10II cells

| | *AuxB1* | *DXR-10II* | *DXR/VCR-10* |
|---|---|---|---|
| Resistance index[a] to | | | |
| VCR | 1.0 (15 ng ml$^{-1}$)[b] | 3.8[c] | 6.0[c,d] |
| ADR | 1.0 (11 ng ml$^{-1}$)[b] | 1.3 | 2.4[d,e] |
| Pgp overexpression[f] | | | |
| C219 | 1.0 | 6.1 ± 0.9 | 12.1 ± 1.0 |
| C494 | 1.0 | 2.7 ± 0.2 | 7.1 ± 0.4 |
| Relative mRNA expression[g] | 1.00 | 1.35 ± 0.1 | 8.00 |
| Pgp half-life | ND | ⩾ 40 h | 20 h |
| Rh123 remaining after 15 min efflux (%) | | | |
| − VRP | 78 ± 9 | 37 ± 4 | 20 ± 1 |
| + VRP (6.6 μM) | 100 ± 1 | 95 ± 1 | 98 ± 1 |

Results represent the means ± s.e.m. [a]Resistance index: ratio of mean $IC_{50}$ values of drug-resistant subline relative to that of AuxB1 cells. [b]$IC_{50}$ values for AuxB1 cells, i.e. the drug concentration required to reduce cell survival to 50% of untreated control. [c]Statistically different from AuxB1 cells, $P < 0.01$. [d]Statistically different from DXR-10II cells, $P < 0.01$. [e]Statistically different from AuxB1 cells, $P < 0.05$. [f]As determined by comparative densitometry of Western blots. [g]As determined by comparative densitometry of Northern blots. ND, not determined.

**Figure 1** Response of AuxB1 (●), DXR-10II (▲) and DXR-10/VCR-10 (▼) cells to a 24 h exposure of (a) VCR and (b) DOX as determined by colony-forming assay. The results represent the means ± s.e.m. of two experiments.

**Figure 2** Expression of Pgp in differentially selected CHO sublines by Western blotting. Crude membrane fractions (50 μg protein per lane) were separated by SDS–PAGE, transferred to nitrocellulose and probed with either (a) C219 or (b) C494 and $^{125}I$-labelled anti-mouse IgG. $CH^RC5$ cells were used as a positive control for Pgp expression. (The lane labelled VRP/DXR-10 refers to another newly developed subline, details of which will be published elsewhere, not relevant to this paper.)

imately 25%) over the first 60 min, while the DXR-10II cells effluxed 60% in the first 15 min. The DXR-10/VCR-10 cells showed a very rapid efflux of Rh123, with 80% being removed from the cells in 15 min. This faster efflux of Rh123, relative to the DXR-10II cells, was consistent with their higher level of Pgp expression. VRP inhibited efflux of Rh123 in all sublines, as demonstrated by the percentage fluorescence remaining in the cells after 15 min in the presence of VRP (Table I), consistent with Pgp-mediated efflux of intracellular fluorescence.

*Turnover of Pgp in the DXR-10/VCR-10 cells*

Since Pgp mRNA increased concomitantly with Pgp overexpression in the DXR-10/VCR-10 cells, Pgp turnover was examined in the DXR-10/VCR-10 cells to investigate whether it occurred at a rate similar to that in other drug-selected CHO cell lines or with the slower half-life observed in DXR-10II cells (McClean *et al.*, 1993*b*). Densitometry of resultant autoradiographs (see Figure 5) revealed that levels of Pgp in DXR-10/VCR-10 cells were reduced to 50% within approximately 20 h, clearly closer to the range observed for the two COL-selected CHO sublines (12 and 17 h) than that noted in the DXR-10II cell Line (≥ 40 h) (McClean *et al.*, 1993*b*).

### Discussion

In the original report (Hill *et al.*, 1990) showing that when AuxB1 cells are exposed to 20 as opposed to ten fractions of X-rays resistance to VCR, COL and VP-16 does not increase concomitantly and Pgp expression is not further enhanced it was suggested that the level of resistance expressed had an upper threshold in these irradiated (DXR-10) cells. This finding contrasts with the more general observation in MDR drug-selective tumour cells: increased resistance to drugs and associated elevations in Pgp and Pgp mRNA occur with increasing selection pressure (Shen *et al.*, 1986; Bradley *et al.*, 1989). We have now shown that when CHO/DXR-10 cells

**Figure 3** Expression of Pgp mRNA in differentially derived CHO sublines determined by Northern analysis of poly(A)$^+$ RNA (2 or 4 µg per lane) prepared from CHO sublines and probed with pCHP1 and GAPDH cDNA probes. CH$^R$C5 cells were used as a positive control.

**Figure 4** Efflux of Rh123 from CHO sublines (●, AuxB1; ▲, DXR-10II; ▼, DXR-10/VCR-10). Cells were preloaded with Rh123 following a 45 min incubation with 200 ng ml$^{-1}$ Rh123 at room temperature. Cells were washed and Rh123 efflux was followed over a 60 min period by flow cytometry. The graph represents the mean ± s.e.m. of two experiments.

**Figure 5** Turnover of Pgp in DXR-10/VCR-10 cells as determined by immunoprecipitation of $^{35}$S-labelled Pgp with C219 monoclonal antibody at different times. Results of two independent experiments are shown as **a** and **b**. C4, CH$^R$C5 cells used as a positive control for Pgp expression; M, molecular weight markers in kDa.

receive ten pulsed exposures to VCR they become more resistant to VCR and their overexpression of Pgp is increased. Furthermore, the increased ability of these DXR-10/VCR-10 cells to efflux Rh123 confirms that there is an increase in functional Pgp expression. The work outlined in this report, therefore, demonstrates that the apparent plateau in drug resistance can be overcome by drug selection but not, as previously reported (Hill *et al.*, 1990), by further X-ray selection.

Certain features of the phenotype associated with the DXR-10II cells are altered as a result of their further drug selection. These DXR-10/VCR-10 cells proved to be resistant to DOX, suggesting that the Pgp specificity of these cells is similar to that of other drug-selected resistant cells (Shen *et al.*, 1986; Bradley *et al.*, 1989), rather than showing the distinctive drug resistance profile of the DXR-10 cells (Hill *et al.*, 1990). The lack of DOX resistance is a characteristic not only of CHO cells that have been exposed to prior X-irradiation (Hill *et al.*, 1990), but also of human tumour sublines pretreated with seven courses of X-irradiation (Hill, 1991), which similarly express a multiple drug resistance phenotype. The development of DOX resistance in these DXR-10/VCR-10 cells therefore implies that the drug resistance phenotype that was induced in these cells following drug selection is modified from that shown following X-ray pretreatments. One possible explanation for this may be that the Pgp that is overexpressed following X-ray pretreatment, although recognised by both C219 and C494 monoclonal antibodies (McClean & Hill, 1993a), differs from the Pgp that is overexpressed following drug selection and exhibits an altered substrate recognition profile. Choi *et al.* (1988) observed that mutations in the *MDR1* sequence which occurred during COL selection of a KB subline resulted in an amino acid change (glycine 185 to valine 185). These KB-8-5-11-24 cells that expressed this mutated Pgp showed preferential resistance to COL over other drugs such as DOX and VBL (Choi *et al.*, 1988). In agreement with this, independent studies have shown that single amino acid substitutions introduced into cells in the eleventh or sixth transmembrane domain of Pgp alter its substrate specificity (Gros *et al.*, 1991; Devine *et al.*, 1992). These altered Pgps confer cross-resistance patterns significantly different from those conferred by the non-mutated protein (Gros *et al.*, 1991; Devine *et al.*, 1992). These findings indicated that the Pgp sequence can determine the pattern of cross-resistance expressed. However, it can be predicted that the frequency of point mutations following a 9 Gy X-ray dose would be of the order of $10^{-5}$ per cell (Thacker, 1992). Therefore, because of the manner in which the DXR-10 cells were derived, with the AuxB1 cells receiving X-ray doses resulting in only a two log cell kill per fraction, and the fact that the surviving population was not cloned (Hill *et al.*, 1990), it is unlikely that single amino acid mutations resulted in Pgp overexpresion throughout the DXR-10 cell population. Nevertheless, it is possible that any alteration in Pgp, including qualitative changes in glycosylation or phosphorylation or even in subcellular distribution, might modulate the ability of Pgp to transport ADR. Future studies will aim to examine these possibilities.

Expression of Pgp in the CHO/DXR-10 cells was associated with a post-translational increase in Pgp stability, rather than with any elevation of Pgp mRNA (McClean *et al.*, 1993*a*), a finding also observed in an X-ray-pretreated human ovarian tumour cell line (McClean & Hill, 1993*b*). Comparative densitometry of Northern blots revealed that Pgp expression in the DXR-10/VCR-10 cells was associated with a 6-fold increase in Pgp mRNA, suggesting that Pgp expression was regulated by transcriptional regulation in drug-selected cells, contrasting with post-translational regulation in cells selected with X-irradiation only. The half-life of Pgp in these DXR-10/VCR-10 cells was found to be 20 h, which was similar to that of the drug-selected CHO sublines rather than that observed in the DXR-10II cells, from which this subline was derived. This appears to confirm that regulation of Pgp expression differs in resistant cells which have been selected with X-rays only, as opposed to those selected with cytotoxic drugs alone or with drugs following X-ray selection.

The precise mechanism(s) involved in the expression of multiple drug resistance following X-ray selection remain to be established. One possibility suggested by the data presented here is that irradiation damages some component involved in the turnover of Pgp. Subsequent further selection with X-irradiation has no further effect since the damage occurred during the initial selection. If this were the case, subsequent selection with VCR would result in increased transcription of *pgp1* together with an extended half-life of Pgp. Since the half-life of Pgp in DXR-10/VCR cells is only 20 h, it appears that the mechanisms involved are rather more complex. It is also interesting to note that Pgp expressed in cells that showed resistance to DOX had a half-life of 12–20 h, while the DXR-10 cells, in which Pgp has been demonstrated to have an extended half-life, did not show any resistance to DOX. While this observation may be fortuitous, a relationship may exist between factors that regulate Pgp stability and the drug resistance profile expressed.

The increased resistance of these DXR-10/VCR cells to DOX may also involve other mechanisms either in association with or independent of Pgp overexpression. Modified glutathione levels and expression of glutathione-related enzymes and certain antioxidant enzymes such as catalase and superoxide dismutase have been implicated in certain DOX-resistant MDR sublines, but this is not a universal finding, as discussed recently (Hosking *et al.*, 1990; McClean *et al.*, 1993*b*). However, our report that none of these parameters was altered significantly in the drug-selected $CH^RC5$ subline, which expressed resistance to DOX and was derived from the same AuxB1 parental cells used in this present study, suggests that they are unlikely to play a significant role in influencing ADR cytotoxicity in these CHO sublines.

In summary, characterisation of a newly derived subline selected for resistance by X-ray and then VCR exposures provides further information on the expression of multiple drug resistance following X-ray pretreatment. These data show that the effect of further drug selection was additive to the expression of multiple drug resistance resulting from the initial X-ray treatment. However, it appears that subsequent drug selection resulted in an additional 'classical' MDR-like phenotype (including elevations in Pgp mRNA, reduced Pgp half-life and resistance to DOX) rather than merely enhancing expression of the specific novel phenotype identified following fractionated X-irradiation pretreatment. These observations, if confirmed in similarly derived human tumour sublines, may have some relevance in designing clinical combined modality therapies, although radiation could alter subsequent chemotherapy response via other pathways, such as damage to tumour vasculature altering drug delivery.

### References

ANDERSON, D.J. & BLOBEL, G. (1983). Immunoprecipitation of proteins from a cell-free translation. *Methods Enzymol.*, **96**, 111–120.

BADLEY, J.E., BISHOP, G.A., ST JOHN, T. & FRELINGER, J.A. (1988). A simple rapid method for the purification of poly $A^+$ RNA. *Biotechniques*, **6**, 114–116.

BIEDLER, J.L. (1992). Genetic aspects of multidrug resistance. *Cancer*, **70**, 1799–1809.

BRADFORD, M.M. (1976). A rapid and sensitive method for the quantitation of microgram quantities of protein utilising the principle of protein-dye binding. *Anal. Biochem.*, **72**, 248–254.

BRADLEY, G., NAIK, M. & LING, V. (1989). P-glycoprotein expression in multidrug resistance human ovarian carcinoma cell lines. *Cancer Res.*, **49**, 2790–2796.

BRUCE, W.R., MEEKER, B.E. & VALERIOTE, F.A. (1966). Comparison of the sensitivity of normal hematopoietic and transplanted lymphoma colony-forming cells to chemotherapeutic agents administered *in vivo*. *J. Natl Cancer Inst.*, **37**, 233–245.

BURTON, K. (1956). A study of the conditions and mechanism of the diphenylamine reaction for the colorimetric estimation of deoxyribonucleic acid. *Biochem. J.*, **62**, 315–323.

CHOI, K., CHEN, C.-J., KRIEGLER, M. & RONINSON, I.B. (1988). An altered pattern of cross-resistance in multidrug-resistant human cells results from spontaneous mutations in the *mdr*1 (P-glycoprotein) gene. *Cell*, **53**, 519–529.

CHURCH, G.M. & GILBERT, W. (1984). Genomic sequencing. *Proc. Natl Acad. Sci. USA*, **81**, 1991–1995.

CORDON-CARDO, C. & O'BRIEN, J.P. (1991). The multidrug resistance phenotype in human cancer. In *Important Advances in Oncology*, De Vita, V.T., Hellman, S. & Rosenberg, S.A. (eds) pp. 19–38. J.B. Lippincott: Philadelphia.

DEVINE, S.E., LING, V. & MELERA, P.W. (1992). Amino acid substitutions in the sixth transmembrane domain of P-glycoprotein after multidrug resistance. *Proc. Natl Acad. Sci. USA*, **89**, 4564–4568.

FAIRBANKS, G., STECK, T.L. & WALLACH, D.F.H. (1971). Electrophoretic analysis of the major polypeptides of the human erythrocyte membrane. *Biochemistry*, **10**, 2606–2617.

GROS, P., DHIR, R., CROOP, J. & TALBOT, F. (1991). A single amino acid substitution strongly modulates the activity and substrate specificity of the mouse mdr1 and mdr3 drug efflux pumps. *Proc. Natl Acad. Sci. USA*, **88**, 7289–7293.

HILL, B.T. (1991). Interactions between antitumour agents and radiation and the expression of resistance. *Cancer Treat. Rev.*, **18**, 149–190.

HILL, B.T., DEUCHARS, K., HOSKING, L.K., LING, V. & WHELAN, R.D.H. (1990). Overexpression of P-glycoprotein in mammalian tumor cell lines after fractionated X-irradiation *in vitro*. *J. Natl Cancer Inst.*, **82**, 607–612.

HOSKING, L.K., WHELAN, R.D.H., SHELLARD, S.A., BEDFORD, P. & HILL, B.T. (1990). An evaluation of the role of glutathione and its associated enzymes in the expression of differential sensitivities to antitumour agents shown by a range of human tumour cell lines. *Biochem. Pharmacol.*, **40**, 1833–1842.

KARTNER, N., EVERDEN-PORELLE, D., BRADLEY, G. & LING, V. (1985). Detection of P-glycoprotein in multidrug resistant cell lines by monoclonal antibodies. *Nature*, **316**, 820–823.

LUDESCHER, C., THALER, J., DRACH, D., DRACH, J., SPITALER, M., GATTRINGER, C., HUBER, H. & HOFMANN, J. (1992). Detection of activity of P-glycoprotein in human tumour samples using rhodamine 123. *Br. J. Haematol.*, **82**, 161–168.

McLEAN, S. & HILL, B.T. (1993*a*). Evidence of post-translational regulation of P-glycoprotein associated with the expression of a distinctive multiple drug resistance phenotype in Chinese hamster ovary cells. *Eur. J. Cancer* **29A**, 2243–2248.

McCLEAN, S. & HILL, B.T. (1993*b*). Post-translational regulation of P-glycoprotein in mammalian tumour cells expressing a distinctive multiple drug resistance phenotype after exposure to fractionated X-irradiation. *Proc. Am. Ass. Cancer Res.*, **34**, 313.

McCLEAN, S., HOSKING, L.K. & HILL, B.T. (1993*a*). Dominant expression of multiple drug resistance after *in vitro* X-irradiation exposure in intraspecific Chinese hamster ovary hybrid cells. *J. Natl Cancer Inst.*, **85**, 48–53.

McCLEAN, S., WHELAN, R.D.H., HOSKING, L.K., HODGES, G.M., THOMPSON, F.H., MEYERS, M.B., SCHUURHUIS, G.J. & HILL, B.T. (1993*b*). Characterisation of the P-glycoprotein overexpressing drug resistance phenotype exhibited by Chinese hamster ovary cells following their *in vitro* exposure to fractionated X-irradiation. *Biochim. Biophys. Acta*, **1177**, 117–126.

RIORDAN, J.R., DEUCHARS, K., KARTNER, N., ALON, N., TRENT, J. & LING, V. (1985). Amplification of P-glycoprotein genes in multi-drug resistant mammalian cell lines. *Nature*, **316**, 817–819.

SAMBROOK, J., FRITSCH, E.F. & MANIATIS, T. (1989). *Molecular Cloning: A Laboratory Manual*, pp. 7.46–7.49. Cold Spring Harbour Press: Cold Spring Harbour, N.Y.

SHEN, D.W., FOJO, A., CHIN, J.E., RONINSON, I.B., RICHERT, N., PASTAN, I. & GOTTESMAN, M.M. (1986). Human multidrug resistant cell lines: increased mdr1 expression can precede gene amplification. *Science*, **232**, 643–645.

THACKER, J. (1992). Radiation-induced mutation in mamalian cells at low doses and low dose rates. In *Advances in Radiation Biology*, Nygaard, O.F., Sinclair, W.K. & Leu, J.T. (eds) pp. 77–121. Academic Press: San Diego.

TSO. J.Y., SUN, X.-H., TU-HO, K., REECE, K.S. & WU, R. (1985). Isolation and characterisation of rat and human glyceraldehyde-3-phosphate dehydrogenase cDNAs: genomic complexity and molecular evolution of the gene. *Nucleic Acids Res.*, **13**, 2485–2502.

WHELAN, R.D.H. & HILL, B.T. (1993). Differential expression of steroid receptors, HSP27 and pS2 in a series of drug resistant breast tumor cell lines derived following exposure to antitumour drugs or to fractionated X-irradiation. *Breast Cancer Res. Treat.*, **26**, 23–39.